# Discovery of Putative Phosphorylation Sites in Cre1 and Ace1 as Engineering Targets for Enhanced Cellulase Production in *Trichoderma reesei*

**DOI:** 10.3390/ijms27209145

**Published:** 2026-10-15

**Authors:** Jiahui Liu, Sunxing Wang, Xin Gao, Yumeng Chen, Wei Wang

**Affiliations:** State Key Lab of Bioreactor Engineering, East China University of Science and Technology, Shanghai 200237, China

**Keywords:** *Trichoderma reesei*, cellulase, carbon catabolite repression, phosphorylation, Cre1, Ace1, combined regulation

## Abstract

In the filamentous fungus *Trichoderma reesei*, cellulase production is subject to carbon catabolite repression (CCR) mediated by the key repressors Cre1 and Ace1. In this study, we identified two previously unreported phosphorylation sites—S204 in Cre1 and S145 in Ace1—through phosphoproteomic profiling. Site-directed mutagenesis revealed that dephosphorylation-mimicking mutations (Cre1S204A and Ace1S145A) significantly relieved CCR and promoted cellulase biosynthesis, while phosphomimetic mutations exerted opposite effects. Notably, the Cre1S204A/Ace1S145A double mutant exhibited a combined enhancement in cellulase activity, achieving a 0.7-fold increase in pNPCase activity compared to the parental strain, along with substantially upregulated cellulase gene transcription and increased extracellular CBH1 accumulation. These results demonstrate that S204 and S145 are functionally relevant phosphorylation sites that negatively modulate cellulase production. Our findings provide novel engineering targets for strain improvement and, more broadly, open new avenues for investigating the phosphorylation-based regulatory mechanisms of Cre1 and Ace1 in future studies. From an applied perspective, the work offers a straightforward and effective genetic strategy for the rational design of high-performance fungal strains for lignocellulose bioconversion.

## 1. Introduction

Bioethanol can be generated from lignocellulosic biomass, a carbon source derived from agricultural byproducts, forest residues, dedicated energy crops, and high-value platform chemicals, serving as a sustainable alternative to fossil fuels [1]. The efficient bioconversion of lignocellulose depends on the synergistic action of various cellulolytic enzymes, including endoglucanases (EGs), cellobiohydrolases (CBHs), and β-glucosidases (BGLs), which collectively hydrolyze cellulose into fermentable sugars [2]. The filamentous fungus *Trichoderma reesei* (*T. reesei*) is the primary industrial chassis for cellulolytic enzyme production as it has remarkable potential for secreting cellulases and hemicellulases [3]. However, its practical application is limited by carbon catabolite repression (CCR), a conserved regulatory mechanism that suppresses cellulase gene expression in the presence of preferred carbon sources [4]. Therefore, overcoming CCR remains a key challenge for enhancing cellulase yield and reducing the cost of lignocellulosic biorefineries.

In *T. reesei*, CCR is governed by a multifaceted transcription factor regulatory network, with Cre1 and Ace1 serving as central repressors [5]. The primary regulator mediating CCR in *T. reesei* is Cre1, a C_2_H_2_-type zinc finger transcription repressor homologous to yeast Mig1 [6]. Cre1 binds to specific DNA motifs (5′-SYGGRG-3′) in the promoters of cellulase genes (e.g., *cbh1* and *eg1*) and silences target gene expression [6,7,8]. The elimination or partial truncation of the Cre1 transcription factor in *T. reesei* significantly relieves CCR, leading to constitutive cellulase production [9]. In the hypercellulase-producing mutant *T. reesei* RUT-C30, Cre1 undergoes a truncation mutation, resulting in a truncated protein, Cre1_96_, which contains only 95 amino acids. Ace1, another critical repressor, contains three Cys_2_His-type zinc fingers. Ace1 binds to the core sequence 5′-AGGCA-3′ and functions by competing with the crucial cellulase transcriptional activator Xyr1 to bind to cellulase gene promoters and interfering with the recruitment of RNA polymerase II [10].

Phosphorylation is a widespread post-translational modification that modulates transcription factor activity, localization, and stability, and it acts as a molecular switch to adapt fungal metabolism to changing carbon environments [11]. Emerging evidence suggests that phosphorylation plays a crucial role in the regulation of CCR in *T. reesei*. For example, phosphorylation of Cre1 at Ser241 affects its nuclear export and degradation, thereby modulating its repressive activity [7]. In addition, phosphorylation at Ser156 enhances the DNA-binding affinity of Cre1, whereas dephosphorylation at this site attenuates CCR [12]. Similarly, a preliminary study suggested that Ace1 is phosphorylated in response to carbon source signals [13]. However, the specific phosphorylation sites and their functional consequences have not yet been identified. Considering the importance of Cre1 and Ace1 in CCR, elucidating the phosphorylation-dependent regulation of these repressors may reveal novel targets for engineering CCR-resistant strains [14]. As such, this study focused on the less-studied S204 site, which lies at the interface of the N-terminal acidic and central repression domains of Cre1, based on the hypothesis that this region acts as a critical conformational switch for fine-tuning CCR [7,14].

Cre1 and Ace1 are global regulators. Direct knockout of these genes markedly reduces the growth rate of fungi [4], since Cre1 and Ace1 act as master controllers of both repressive regulatory networks and essential growth-related pathways. Loss of these regulators causes widespread transcriptional dysregulation of genes involved in glycolysis, the tricarboxylic acid cycle, cell wall assembly, and hyphal development, resulting in insufficient energy supply, defective cell structure, and severely restricted vegetative growth. In addition, cellulase expression is not necessarily enhanced, and cellulase production may decrease in some cases [15]. Therefore, investigating the phosphorylation-mediated regulation of Cre1 and Ace1 and constructing mutants to relieve cellulase repression may be an effective strategy for enhancing cellulase expression. Our phosphoproteomic analysis directly identified Ser204 in Cre1 and Ser145 in Ace1 as high-confidence phosphorylation sites that also exhibit strong evolutionary conservation and localization within functionally critical domains. To determine their functions, we generated phospho-deficient (alanine substitution [A]) and phosphomimetic (aspartic acid [D]) mutants of Cre1 S204 and Ace1 S145, and systematically evaluated their effects on cellulase production in *T. reesei*.

In this study, we aimed to assess the functional roles of key phosphorylation sites in Cre1 (S204) and Ace1 (S145) and elucidate their combined regulatory mechanisms in controlling cellulase expression in *T. reesei*. Our findings provide insights into the molecular basis of fungal CCR and identify novel targets for the rational design of high-yield *T. reesei* strains, thereby advancing industrial biorefining applications.

## 2. Results

### 2.1. Phosphoproteomics Reveals New Phosphorylation Sites

**Figure 1:**
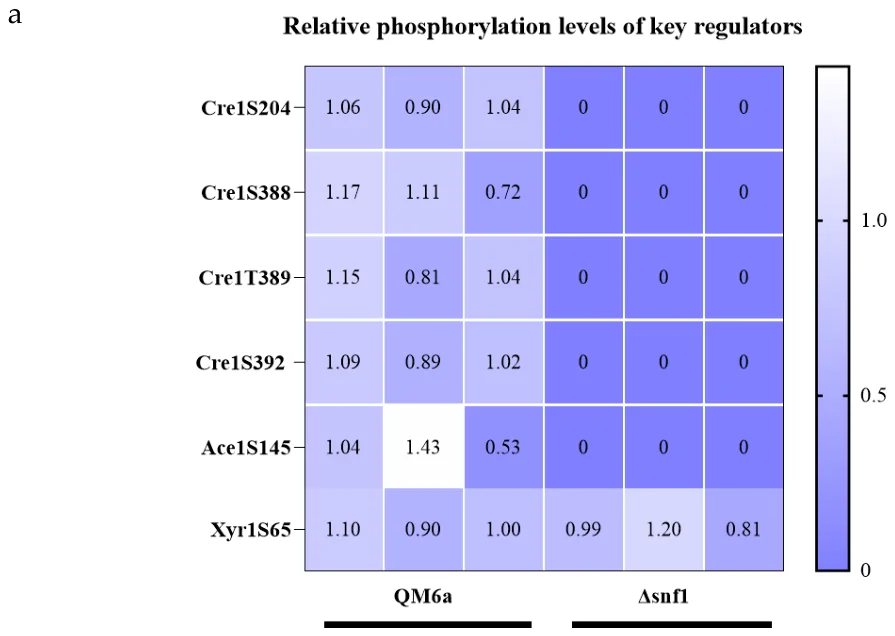

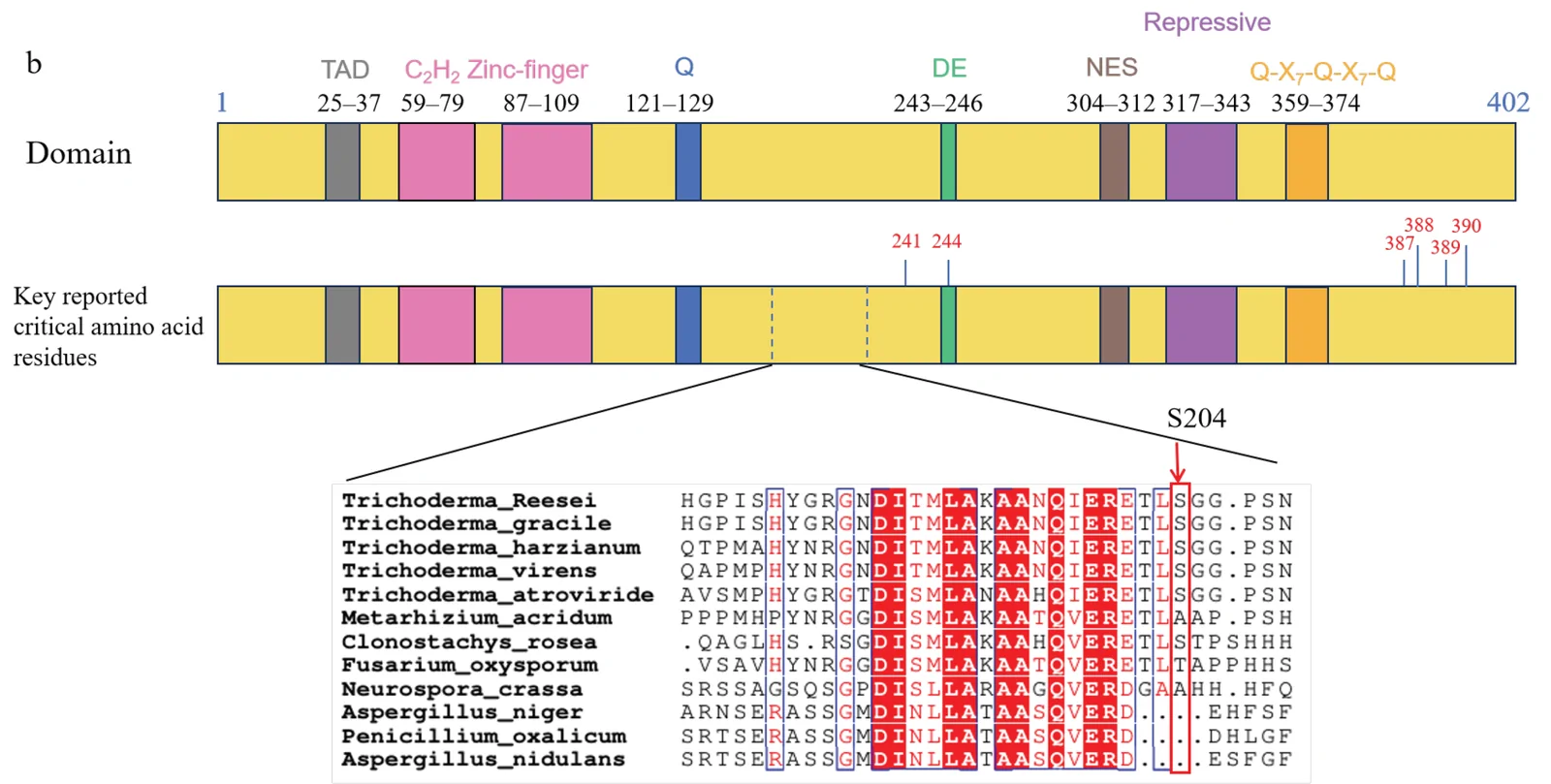

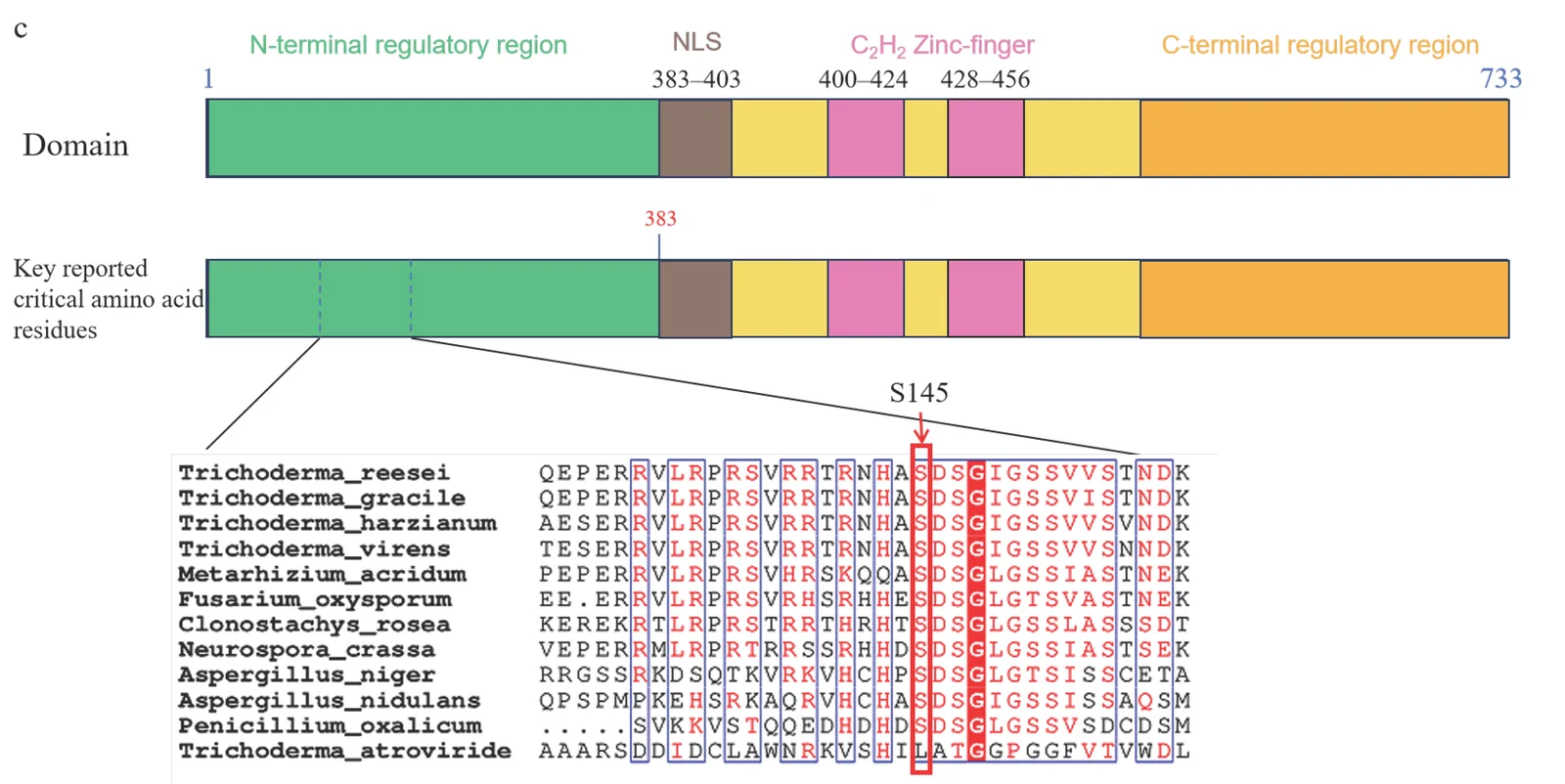
Relative phosphorylation levels of key cellulolytic transcriptional regulators, protein domain architectures, functionally characterized critical amino acid residues, and multispecies evolutionary conservation. Evolutionary conservation analysis of Cre1 and Ace1 was performed across an identical panel of 12 representative filamentous fungal species (*Trichoderma reesei*, *Trichoderma gracile*, *Trichoderma harzianum*, *Trichoderma virens*, *Trichoderma atroviride*, *Metarhizium acridum, Clonostachys rosea*, *Fusarium oxysporum*, *Neurospora crassa*, *Aspergillus niger*, *Penicillium oxalicum*, and *Aspergillus nidulans*). (**a**) Heatmap illustrating the relative phosphorylation levels of Cre1 (S204, S388, T389, and S392), Ace1 (S145), and Xyr1 (S65) residues in the QM6a parental and Δsnf1 mutant strains [18]. The color gradient scale represents relative phosphorylation levels ranging from 0 to 1.4, with 0 indicating no detectable signal. (**b**) Bioinformatic domain prediction was performed for Cre1. Numerals correspond to amino acid (a.a.) coordinate positions, whereas the pink shaded boxes denote the characterized C_2_H_2_ zinc finger motifs, transcription activation domain (TAD; gray), nuclear export signal (NES; brown), DE domain (aspartate–glutamate-rich acidic domain; green), Q-rich region (blue), repressive domain (purple), and Q-X_7_-Q-X_7_-Q motif (orange). Key amino acid sites are highlighted in red. Multiple sequence alignment of the Cre1 S204 residue was performed across *T. reesei*, *T. gracile*, *T. harzianum*, *T. virens*, *T. atroviride*, *M. acridum, C. rosea*, *F. oxysporum*, *N. crassa*, *A. niger*, *P. oxalicum*, and *A. nidulans*. The highly conserved S204 residue is highlighted in red. (**c**) Schematic diagram of Ace1 functional domain architecture containing an N-terminal regulatory region, a conserved sequence that mediates nuclear translocation (NLS), C_2_H_2_ zinc-finger domains, and a regulatory domain located at the C-terminus, with localization of documented key critical residues. Multiple sequence alignment of the Ace1 S145 residue among the above-mentioned 12 representative filamentous fungal species, with the conserved S145 residue highlighted in red.

The energy-sensing kinase Snf1 has been implicated in the regulation of cellulase production in T. *reesei*, as its overexpression partially restores cellulase biosynthesis impaired by Rab7 knockdown under cellulose-inducing conditions [16]. Previous findings have confirmed that *T. reesei* Snf1 mediates the phosphorylation of yeast CCR Mig1 but not fungal Cre1 [17]. This post-translational modification suggests a putative regulatory strategy for alleviating CCR and relieving the suppression of cellulase gene expression. Our previously published data [18] demonstrated that Snf1 deletion resulted in a near-complete loss of phosphorylation at Ser204, 388, 389, and 392 in Cre1 and Ser145 in Ace1 (Figure 1a and Table S1), with both sites exhibiting robust phosphorylation signals in the parental Qa7.5 strain. Although previous studies have identified the functional significance of Cre1 residues S388, S389, and S392 in *T. reesei* [14], our findings provide novel mechanistic insights by demonstrating that these sites may be direct or indirect targets of Snf1-mediated phosphorylation, thereby mediating downstream regulatory activity. Furthermore, we identified two previously unreported phosphorylation sites: S145 in Ace1 and S204 in Cre1 (Figure 1b,c). In this study, we successfully mapped the phosphorylation site located on Ace1 for the first time. Given their unique status as novel regulatory elements, these residues represent compelling targets for future functional characterization to elucidate their specific roles in Snf1–Cre1 and Snf1–Ace1 signaling pathways.

Cre1 is the master regulator of CCR in *T. reesei*; therefore, we assessed the functional significance of Cre1 Ser204 phosphorylation. Mutation of this residue to alanine (mimicking constitutive dephosphorylation) significantly increased the cellulase yield, which directly demonstrates the importance of phosphorylation at Cre1 Ser204 for the repression of cellulase gene expression. Ace1 acts as an auxiliary regulator of CCR [4]. We hypothesized that mimicking dephosphorylation at Ser145 would exert effects similar to those of Cre1 Ser204 dephosphorylation in relieving CCR and boosting cellulase production.

In contrast, phosphorylation of XYR1 (the master transcriptional activator of cellulase genes) at Ser65 was unaffected by *snf1* deletion (Figure 1a), indicating that Snf1 does not regulate the activity of XYR1 by modulating its phosphorylation level at this site.

### 2.2. Cre1 S204 Phosphorylation Represses Cellulase Production in T. reesei

**Figure 2:**
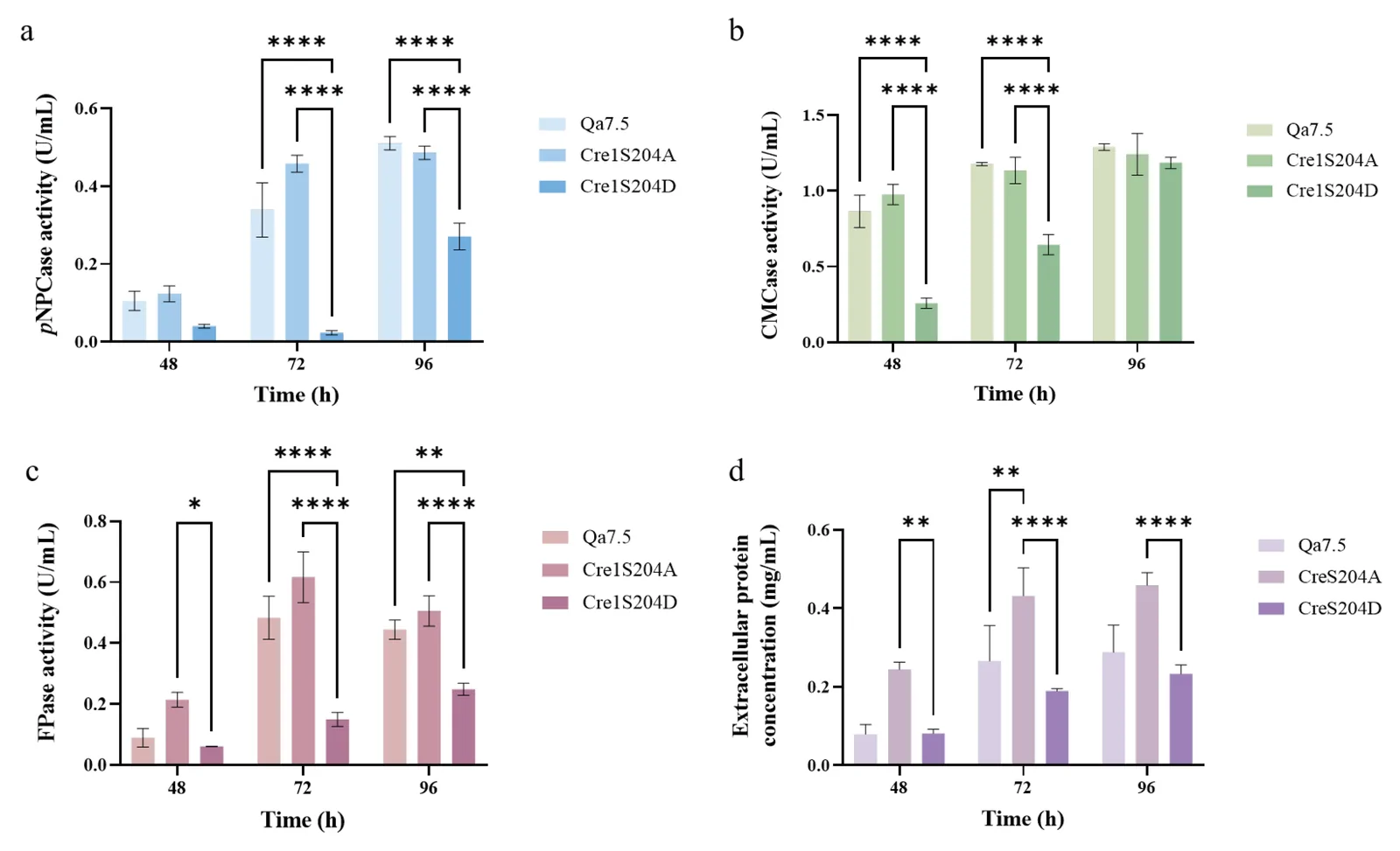
Effects of Cre1 S204 mutations on cellulase activity and extracellular protein levels in *Trichoderma reesei*. (**a**) Cellobiohydrolase (pNPCase) activity. (**b**) Endoglucanase (CMCase) activity. (**c**) Hydrolase (FPase) activity. (**d**) Extracellular protein concentrations. Assays were performed in triplicate (mean ± standard deviation). **** *p* < 0.0001, ** *p* < 0.01, and * *p* < 0.05 (one-way analysis of variance, followed by Tukey’s test).

To assess the functional role played by Cre1 S204 phosphorylation in regulating cellulase biosynthesis, we quantified key extracellular enzymes (pNPCase, CMCase, and FPase) and total protein levels in *T. reesei* strains harboring S204 mutations (Cre1S204A and Cre1S204D) and compared them with those in the parental strain Qa7.5 (Figure 2). First, we assessed pNPCase activity (Figure 2a), a surrogate for CBH (e.g., Cel6A/CBH2) function. The phosphomimetic mutant Cre1S204D exhibited significantly reduced pNPCase activity at all time points (24–120 h), showing a 42–58% decrease in activity compared to Qa7.5 at 72–120 h. In contrast, the pNPCase activity of the dephosphorylation-mimetic mutant Cre1S204A was 18–25% higher than that of Qa7.5, particularly at 72 h (*p* < 0.01). A consistent regulatory pattern was observed for CMCase activity (Figure 2b), which reflects EG function. At 72 h, Cre1S204D showed a marked 63% reduction in CMCase activity compared with Qa7.5 (**** *p* < 0.0001), whereas Cre1S204A maintained 21% higher activity than the parental strain (*p* < 0.01). The total cellulolytic capacity, as measured by FPase activity (Figure 2c), followed an identical trend:

Cre1S204D exhibited a 57% decrease in FPase activity compared with Qa7.5 at 72 h (**** *p* < 0.0001), whereas Cre1S204A showed no significant difference or a modest 12% enhancement relative to Qa7.5. These enzymatic changes were further corroborated by extracellular protein concentration measurements (Figure 2d). Consistent with the enzyme activity data, Cre1S204D accumulated 41% less extracellular protein than Qa7.5 at 72 h (*p* < 0.01), whereas Cre1S204A showed a 28% increase in protein secretion compared to the parental strain (*p* < 0.01). Collectively, these results suggest that phosphomimetic mutations at S204 (Cre1S204D) strongly repress cellulase production, whereas abolishing phosphorylation (Cre1S204A) alleviates this repression. In addition, these findings suggest that S204 phosphorylation is a critical regulatory switch that modulates Cre1-mediated CCR of cellulase gene expression in *T. reesei*.

### 2.3. Transcriptomic Analysis of the Cre1 S204 Mutant

**Figure 3:**
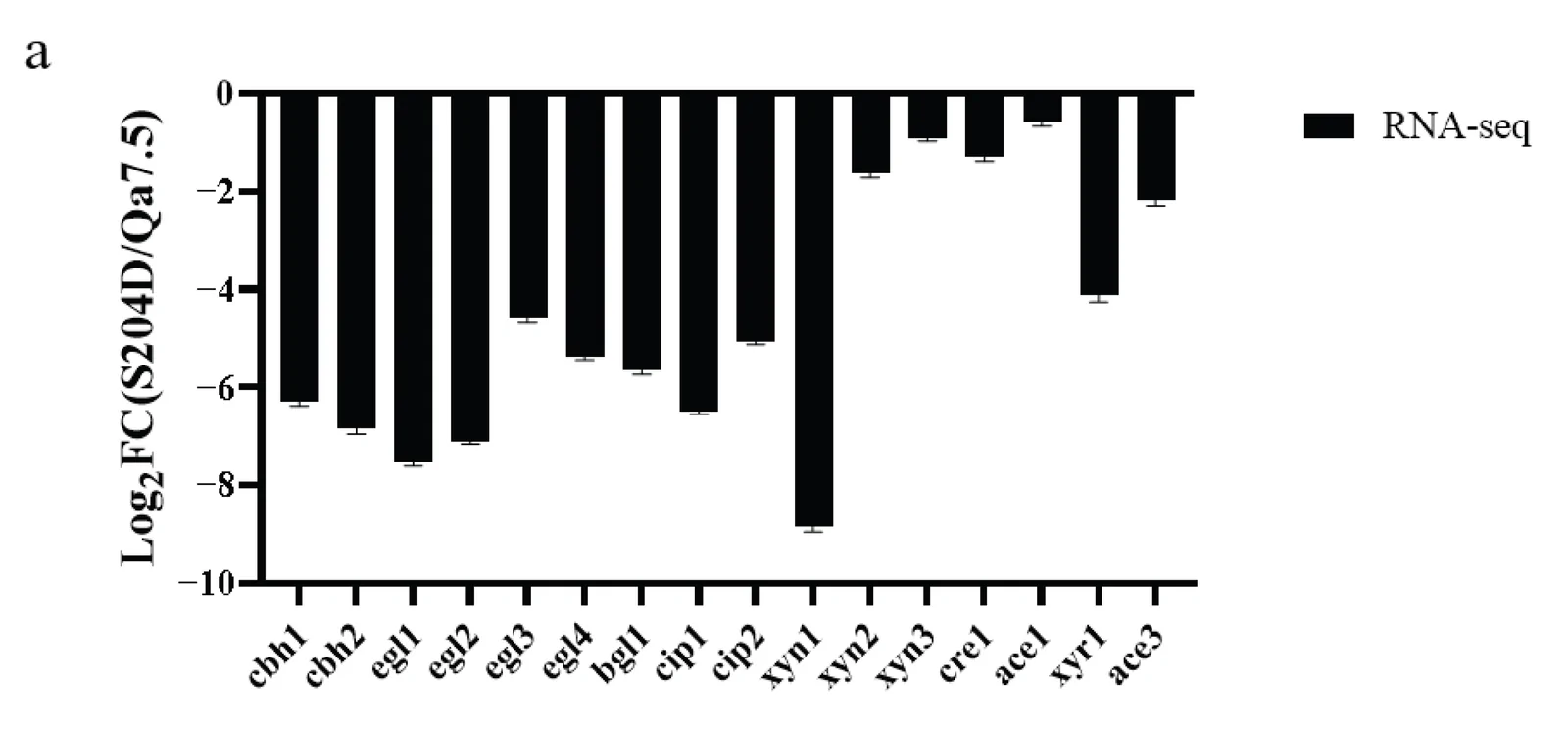

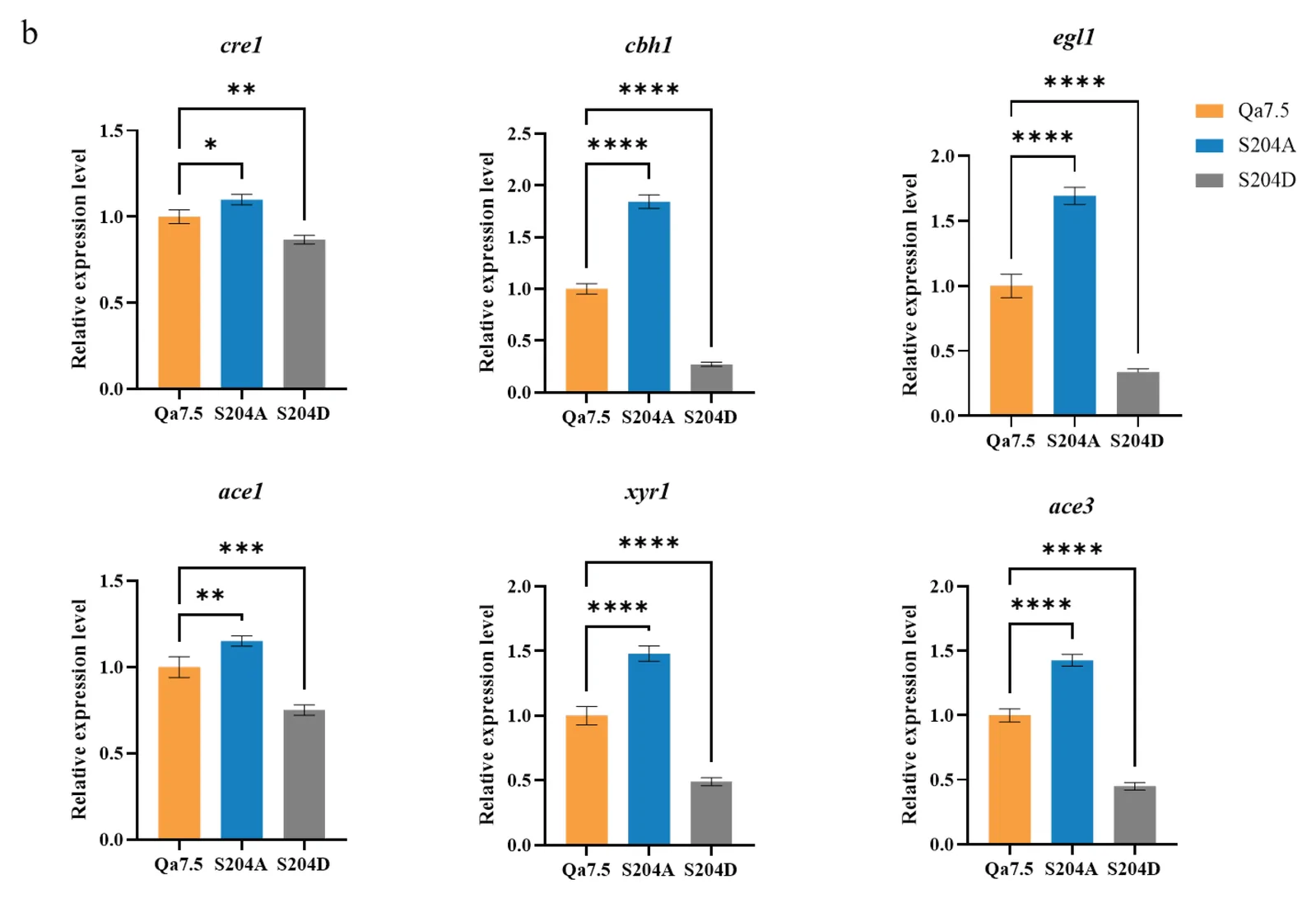
Effects of Cre1 S204 phosphorylation-site mutations on the transcription of cellulase-related genes in *Trichoderma reesei*. (**a**) Log2 fold change (log_2_FC) in cellulolytic and hemicellulolytic gene expression levels in the Cre1S204D mutant strain in comparison with the Qa7.5 parental strain, as determined via RNA-sequencing analysis. (**b**) Relative transcript levels of cellulase-related regulatory genes (*ace1*, *cre1*, *xyr1*, and *ace3*) and cellulase genes (*egl1* and *cbh1*) in Qa7.5, S204A, and S204D strains. Transcript levels were determined by RT-qPCR, normalized to *sar1*, with Qa7.5 used as the calibrator. Data are presented as the mean ± standard deviation (SD) from three independent biological replicates. Statistical significance was determined using Student’s *t*-test. For each gene, the transcript levels in the S204A and S204D strains were compared with those of the same gene in Qa7.5. **** *p* < 0.0001, *** *p* < 0.001, ** *p* < 0.01, and * *p* < 0.05.

Cellulolytic and xylanolytic enzymes are central to the lignocellulose-degrading capacity of filamentous fungi, and they enable the efficient breakdown of cellulose and hemicellulose into fermentable sugars; as such, they are essential to the overall biomass bioconversion efficiency. The relative transcriptional levels of key cellulases (*cbh1*, *cbh2*, *egl1*, *egl2*, *egl3*, *egl4*, *bgl1*, *cip1*, and *cip2*), xylanases (*xyn1*, *xyn2*, and *xyn3*), transcriptional repressors (*cre1* and *ace1*), and activators (*xyr1* and *ace3*) were compared between the Cre1S204D mutant (S204D) and control strain Qa7.5. RNA sequencing confirmed the repressive effect of Cre1 S204 phosphorylation on cellulase gene expression (Figure 3). Most cellulases and xylanases showed a marked decrease in transcription (approximately 4–256-fold), consistent with reduced cellulase activity (Figure 3).

Transcriptomic analysis comparing the Cre1S204D mutant with the parental Qa7.5 strain revealed distinct regulatory effects on key transcription factors governing glycoside hydrolase expression. Notably, *ace1* transcription remained largely unchanged, whereas *cre1* was significantly downregulated, indicating that the S204D substitution modulated target enzyme gene expression and exerted autoregulatory effects on its transcription. The crucial transcriptional activators, *xyr1* and *ace3*, were markedly repressed by 17.15- and 4.53-fold, respectively. This strong downregulation of the two master activators represents a key molecular mechanism underlying the observed reduction in the transcription and enzymatic activity of cellulases and xylanases in the S204D mutant.

### 2.4. Cre1–Ace1 Double Mutants Exhibit Combined Enhancement and Superimposed Repression of Cellulase Synthesis

**Figure 4:**
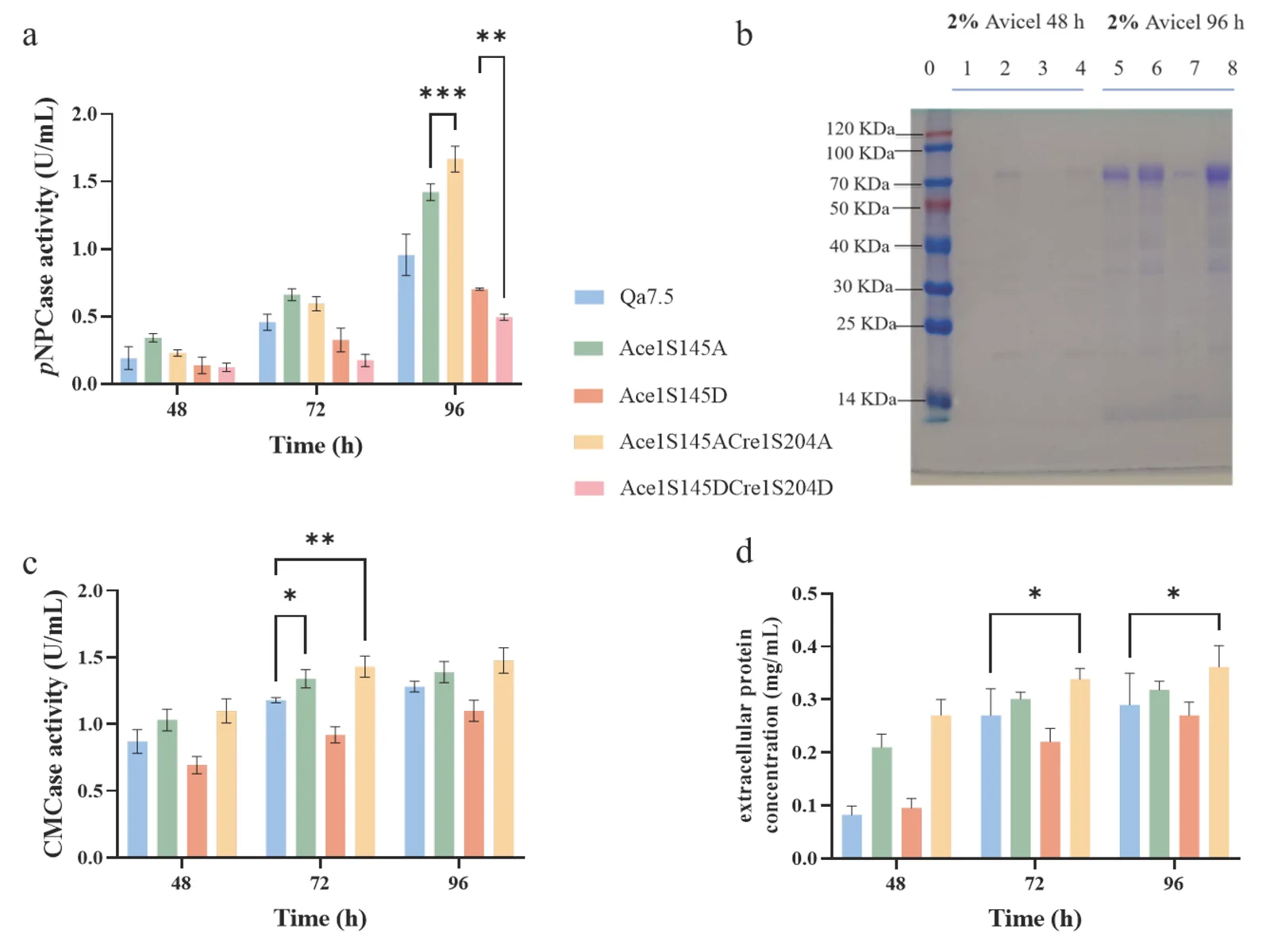
Quantitative determination of cellulase activity combined with SDS-PAGE characterization in Cre1 S204 and Ace1 S145 mutant strains. (**a**) *p*NPCase activity of *T. reesei* single/combined mutant strains at 48, 72, and 96 h. (**b**) SDS-PAGE analysis of extracellular protein levels in strains induced with 2% (*w*/*v*) Avicel at 48 and 96 h. Lanes 1–4: 48 h samples of Qa7.5, Cre1S204A, Cre1S204D, and Ace1S145A–Cre1S204A, respectively; lanes 5–8: 96 h samples of Qa7.5, Cre1S204A, Cre1S204D, and Ace1S145A–Cre1S204A, respectively. Lane 0 represents the protein molecular weight marker. (**c**) CMCase activity of *T. reesei* single and combined mutant strains at 48, 72, and 96 h. (**d**) FPase activity of *T. reesei* single and combined mutant strains at 48, 72, and 96 h. All enzyme activity assays were performed in triplicate, and error bars represent standard deviations. Statistical significance was determined via one-way analysis of variance, followed by Tukey’s post hoc test (*** *p* < 0.001, ** *p* < 0.01, and * *p* < 0.05).

As the predominant cellulase secreted by *T. reesei*, Cel7a undergoes rigorous transcriptional control to regulate its expression. The cel7a gene is controlled by multiple transcription factors, including activators Xyr1, Crz1, Ace3, and Azf1 and repressors Rce1 and Rce2 [19,20,21,22,23,24,25]. In addition, Cre1 and Ace1 act additively to enhance the cellulase activity. To explore the additive regulation of cellulase production by Ace1 and Cre1, we analyzed the *p*NPCase activity and extracellular protein profiles in the single- and double-mutant strains (Figure 4 and Figure S1). As shown in Figure 4a, the double-mutant strain Ace1S145A/Cre1S204A exhibited significantly higher *p*NPCase activity than the parental strain Qa7.5 at all time points, reaching its highest level at 96 h (**** *p* < 0.0001 and ** *p* < 0.01). In contrast, the phosphomimetic strains (Ace1S145D and Ace1S145D/Cre1S204D) showed reduced *p*NPCase activity, consistent with the repressive effects of phosphorylation at these sites. Sodium dodecyl sulfate–polyacrylamide gel electrophoresis (Figure 4b) confirmed that the Ace1S145A/Cre1S204A double mutant accumulated higher levels of extracellular CBH1 (the primary contributor to *p*NPCase activity) than the Qa7.5 and Cre1S204D strains at both 36 and 72 h.

To investigate whether these phosphorylation sites participate in CCR and to define their functional association with Cre1 and Ace1, we evaluated the effects of the mutations on 2-deoxy-D-glucose (2-DG) resistance. The parental strain Qa7.5, the Cre1 mutant, the Ace1 mutant, and the Cre1/Ace1 double mutant were examined for growth on MM plates containing CMCNa and 2-DG. As shown in Figure S2, phospho-ablative (dephosphorylation-mimicking) mutations markedly enhanced resistance to 2-DG, with the double mutation exerting an even more pronounced effect, whereas phosphomimetic (phosphorylation-mimicking) mutations had the opposite effect (Figure S2). These results suggest that the simultaneous abolition of phosphorylation at Ace1 S145 and Cre1 S204 collectively relieved CCR and enhanced cellulase expression in *T. reesei*.

## 3. Discussion

In this study, we identified two novel functionally critical phosphorylation sites that act as key regulatory nodes within the cellulase transcriptional regulatory network of the filamentous fungus *T. reesei*: S204 in Cre1 and S145 in Ace1. Cre1 is a canonical carbon catabolite repressor that dominates metabolic flux redistribution during lignocellulose degradation, and Ace1 is a well-characterized transcriptional repressor that specifically targets cellulase and xylanase gene promoters. Both are central to the fine-tuning of hydrolytic enzyme expression. Our findings underscore the functional relevance of post-translational modifications, particularly phosphorylation, in the regulation of the balance between cellulase synthesis and carbon metabolism in filamentous fungi.

S204 and S145 were identified by phosphoproteomic profiling of the Δsnf1 mutant strain. As a conserved serine/threonine kinase and central regulator of carbon catabolite derepression, AMPK/Snf1 plays an indispensable role in sensing extracellular carbon source availability, transducing metabolic signals, and mediating the phosphorylation of downstream transcription factors to coordinate adaptive responses, including cellulase production [16,26]. Our observation that snf1 deletion drastically reduces the phosphorylation levels of S204 (Cre1) and S145 (Ace1) reveals a novel regulatory mode whereby Snf1 modulates cellulase expression via direct or indirect phosphorylation of Cre1 and Ace1. These findings expand the spectrum of phosphorylation events associated with Snf1 signaling in filamentous fungi and uncover a new regulatory cascade linked to cellulase gene repression. Given that Ace1 activity is regulated by arginine methylation at R383 [27], the identification of the Snf1-dependent S145 phosphorylation site provides an additional post-translational mechanism for fine-tuning Ace1 function.

Notably, our study corroborates the findings of Han et al. [14] regarding Cre1 phosphorylation. We confirmed the presence of clustered C-terminal phosphorylation sites (S388, T389, and S392) in *T. reesei* Cre1, which have been implicated in the repressive function of Cre1 during CCR. Consistent with the results reported by Han et al. [14], we achieved enhanced cellulase production by mimicking dephosphorylation at these phosphorylation sites.

Phosphorylation is a well-documented modulator of the subcellular localization of transcription factors, typically via regulation of the accessibility of nuclear import/localization signals or alteration of protein–protein interactions. However, because Cre1 harbors a functional nuclear export signal (aa 304–312) and Ace1 contains a conserved nuclear localization signal (aa 383–403), it remains unclear whether the S204D mutation, which mimics constitutive phosphorylation, alters the nuclear-cytoplasmic shuttling dynamics of Cre1, thereby directly affecting its ability to bind to target gene promoters and exert transcriptional repression. Addressing this question using complementary approaches, including fluorescence microscopy (e.g., green fluorescent protein-tagged Cre1 localization) and chromatin immunoprecipitation assays, may provide more comprehensive insights into the mechanisms by which phosphorylation spatiotemporally regulates Cre1 and Ace1 functions. However, fusion with fluorescent proteins may have unpredictable effects on the native functions of Cre1 and Ace1. In addition, obtaining sufficiently strong fluorescence signals often requires overexpression of the fusion proteins, which may further alter their abundance, intracellular behavior, or regulatory functions and thereby introduce additional experimental bias [28]. Additionally, future studies should explore whether S204D and S145 phosphorylation affect the DNA-binding affinity of Cre1 and Ace1 or their interactions with other regulatory proteins to elucidate the regulatory roles of the newly identified sites. However, this would require prior antibody generation and validation, which is time-consuming.

From a broader biological perspective, the identification of these two phosphorylation sites reveals that *T. reesei* employs convergent phosphorylation-dependent repression through multiple transcription factors to efficiently suppress cellulase expression when preferred carbon sources are available. This layered regulatory strategy likely enables rapid and sensitive metabolic adaptation, reflecting an evolutionarily advantageous mechanism for fungal survival in competitive ecological niches.

Dephosphorylation-mimicking mutations by replacing serine with alanine at positions Cre1-S204 and Ace1-S145 markedly enhanced cellulase production (Figure 2a and Figure 4a). The combined substitution of Cre1-S204A and Ace1-S145A (mimicking dephosphorylation) additively enhanced cellulase production, surpassing single-site mutation effects. This synergism underscores the additive regulatory roles of S204 and S145 in the alleviation of repressive signals, thereby providing a promising strategy for rational strain engineering. Additional mechanistic depth can be determined by probing how phosphorylation modifications alter the subcellular distribution, DNA-binding capacity, and intermolecular interactions of this transcription factor.

Despite these novel insights, several limitations of the current study should be acknowledged. Our data establish phenotypic and correlative links between phosphorylation and cellulase regulation, but direct biochemical evidence demonstrating how phosphorylation at S204 and S145 modulates protein localization, DNA-binding activity, or complex formation is still lacking. Consequently, some mechanistic interpretations remain inferential rather than conclusively proven.

## 4. Materials and Methods

### 4.1. Microbial Strains and Culture Conditions

Recombinant DNA manipulation was performed using the E. coli DH5α strain and fungal genetic transformation was mediated by *Agrobacterium tumefaciens* strain GV3101, as described in the previous literature [29]. Luria–Bertani medium was used for bacterial cultures. *T. reesei* Qa7.5 [23], a mutant of the wild-type strain QM6a, was used as the host strain for protein expression and as the control strain in subsequent experiments. Mandel’s (2× MA) medium [22] containing 2% (*w*/*v*) Avicel (PH-101; Sigma-Aldrich, St. Louis, MO, USA) was used as the culture solution for *T. reesei* liquid fermentation. Mature conidia of *T. reesei* were collected from potato dextrose agar (PDA) plates [21] at 28 °C for 5–7 days until complete conidiation.

### 4.2. Construction of Plasmids and Mutants

To construct a series of Cre1 and Ace1 mutant strains, the upstream and downstream flanking sequences of the native *cre1* and *ace1* genes were amplified from the genomic DNA of *T. reesei* Qa7.5, which served as homologous recombination arms. For *cre1*, three pairs of specific primers (Cre1-1/Cre1S204A-1R, Cre1S204A-2F/Cre1-2, and Cre1-3/Cre1-4) were used to amplify the two upstream (left recombination arm) and one downstream (right recombination arm) fragments. The remaining homologous recombination fragment was directly inserted into the PacI/XbaI multiple cloning sites of the plasmid LML2.1 [22] to yield the intermediate vector pcre1S204A-F. Subsequently, the right recombination arm was inserted into the SwaI locus of pcre1S204A-F to generate the final recombinant plasmid, pcre1S204A (Figure S1). Restriction digestion and Sanger sequencing confirmed the accurate assembly of the target construct.

The pcre1S204A plasmid was introduced into wild-type *T. reesei* Qa7.5 via *Agrobacterium*-mediated conjugation transformation. The cre1S204A mutant strain was obtained by double-crossover homologous recombination between the upstream and downstream recombination arms and genomic DNA (Figure S3). The hygromycin B-resistance marker in this mutant strain was successfully restored by homologous recombination [25].

Recombinant plasmids pcre1S204D, pace1S145A, and pace1S145D were constructed using the same strategy (Figure S3), and the corresponding mutant strains were generated using the same homologous recombination method.

Following *Agrobacterium*-mediated transformation, the resulting transformants were identified and confirmed by colony PCR and DNA sequencing according to a previously described protocol [22]. All the constructed plasmids and generated mutant strains are summarized in Table S2. Oligonucleotide primers used for vector construction and mutant validation are listed in Table S3. Mutants (TrCre1S204A, TrCre1S204D, TrAce1S145A, and TrAce1S145D) were confirmed by DNA sequencing to identify the desired base substitutions.

Double mutants (TrAce1S145DCre1S204D and TrAce1S145ACre1S204A) were generated by repeating the mutagenesis procedure sequentially in the desired parental strain.

### 4.3. Assays for Enzyme Activity and Protein Quantification

For shake-flask fermentation, 40 mL of 2×MA medium [24] containing 2% (*w*/*v*) Avicel as the sole carbon source was inoculated with 2 × 10^6^ conidia, followed by incubation at 28 °C with shaking at 220 rpm for 5 days in 250-mL flasks. Cultures were then harvested at designated time points (48 h, 72 h, and 96 h), followed by centrifugation at 12,000 rpm for 10 min at 4 °C. Supernatants were collected and used for cellulase activity detection and protein concentration measurements, whereas harvested mycelia were used for biomass determination or RNA extraction. Biomass was estimated from intracellular protein content as previously described [30]. Harvested mycelia were treated with 1 M NaOH for 2 h, and intracellular protein was quantified using a modified Lowry assay after centrifugation. Medium-derived background protein was corrected using uninoculated controls, and dry biomass was calculated assuming 0.32 g of intracellular protein per gram of dry cell mass. The total extracellular cellulase activity was assessed as filter paper hydrolase (FPase) activity using a 3,5-dinitrosalicylic acid assay. Endoglucanase (CMCase) and cellobiohydrolase (*p*NPCase) activity were determined. All enzyme activities were measured according to a previously published protocol [21,31,32,33]. One unit of enzymatic activity was defined as the amount of enzyme required to release 1 μmol of reducing sugar or *p*-nitrophenol per minute. The protein content of the fermentation supernatant was determined using a commercial protein assay kit (Bio-Rad, Hercules, CA, USA) based on absorbance detection at 595 nm [30].

### 4.4. Transcriptome Analysis

Mycelial samples were harvested after 72 h of incubation in the MA medium containing 2% (*w*/*v*) Avicel cellulose. Total RNA was extracted according to the manufacturer’s instructions (#RC401–01; Vazyme, Nanjing, China). RNA concentration and purity were quantified on a NanoDrop 2000 spectrophotometer (Thermo Fisher Scientific, Wilmington, DE, USA). Agarose gel electrophoresis was used to examine RNA integrity, and an Agilent 5300 system (Agilent Technologies, Santa Clara, CA, USA) was used to calculate the corresponding RQN indices. Library sequencing was performed on an Illumina NovaSeq X Plus platform using a cBot system (Illumina, San Diego, CA, USA). It was constructed from six RNA samples, including three biological replicates of strain Qa7.5 and three biological replicates of the mutant strain S204D, by Majorbio Ltd. (Shanghai, China). Raw RNA-seq data have been deposited in the SRA database (accession number PRJNA1474866) and are publicly accessible (https://www.ncbi.nlm.nih.gov/). Raw sequencing reads were quality-filtered using fastp. Adapter sequences were removed, low-quality bases at the 3’ ends of reads with quality scores below 20 were trimmed, reads containing more than 10% ambiguous nucleotides (N) were removed, and reads shorter than 20 bp after adapter removal and quality trimming were discarded. The resulting clean reads were aligned to the *Trichoderma reesei* reference genome (GCA000167675v2; Ensembl Fungi, https://fungi.ensembl.org/Trichoderma_reesei/Info/Index; accessed on 1 July 2026) using HISAT2. Gene expression abundance was quantified using RSEM and normalized as transcripts per million (TPM). Differential expression analysis was performed using DESeq2 based on raw read counts. *p* values were adjusted for multiple testing using the Benjamini–Hochberg false-discovery-rate procedure. Genes with |log2FC| ≥ 1 and Padj < 0.05 were considered significantly differentially expressed genes (DEGs).

### 4.5. Phosphoproteomics

*T. reesei* wild-type strain QM6a and the Δsnf1 mutant were incubated in glucose MA medium for 36 h prior to induction with 2% (*w*/*v*) lactose for 12 h. Mycelia from three biological replicates were sampled and submitted to APTBIO (Shanghai, China). Data-independent acquisition mass spectrometry was used for proteomic measurements [18]. The mass spectrometry proteomics and phosphoproteomics data have been deposited in the iProX Consortium (https://www.iprox.org) with the dataset identifier IPX0017803001.

### 4.6. Statistical Analyses

All experiments were performed in triplicate. Error bars represent the mean values ± standard deviation (SD) from three independent determinations. Statistical significance was determined via one-way analysis of variance, followed by Tukey’s post hoc test (**** *p* < 0.0001, *** *p* < 0.001,** *p* < 0.01, and * *p* < 0.05).

### 4.7. Reverse Transcription Quantitative PCR

Reverse transcription quantitative PCR (RT-qPCR) was performed as previously described [19]. Gene expression levels were normalized to *sar1*. Three biological replicates were analyzed, each with three technical replicates. Primer sequences are listed in Table S4.

### 4.8. Sensitivity Growth Assays

Approximately 1.5 μL of 10^4^/mL conidial suspensions from Qa7.5 and mutant strains were spotted on MM solid medium [25] containing either 1% (*w*/*v*) CMCNa or 1.5 mM 2-DG as the carbon source and then incubated at 28 °C for 3 days.

## 5. Conclusions

In summary, we identified two novel phosphorylation sites that serve as pivotal regulatory nodes in the cellulase transcriptional network of *T. reesei*: S204 in the carbon catabolite repressor Cre1 and S145 in the cellulase-specific repressor Ace1. Phosphomimetic mutations (Cre1S204D and Ace1S145D) enhanced the repressive functions of Cre1 and Ace1, significantly reducing cellulase activity and down-regulating cellulase/hemicellulase gene transcription. In contrast, dephosphorylation-mimicking mutations (Cre1S204A and Ace1S145A) reduced CCR and promoted cellulase biosynthesis. The combined substitution of Cre1-S204A and Ace1-S145A (mimicking dephosphorylation) additively enhanced cellulase production, surpassing single-site mutation effects. Collectively, this study focused on the coordinated post-translational regulation of Cre1 and Ace1 by Snf1 for alleviating CCR and boosting cellulase production in *T. reesei*. Moreover, the identified phosphorylation sites and combinatorial mutations may serve as valuable targets for optimizing the production of lignocellulosic biomass-degrading enzymes.

## Data Availability

All data generated/analyzed in this study are included in the article or Supplementary Materials. Further inquiries can be directed to the corresponding author.

## Supplementary Materials

The following supporting information can be downloaded at: https://www.mdpi.com/article/10.3390/ijms27209145/s1.

## Abbreviations

The following abbreviations are used in this manuscript:

AMPK	AMP-activated protein kinase
CBH	Cellobiohydrolase
CCR	Carbon catabolite repression
SDS-PAGE	Sodium dodecyl sulfate–polyacrylamide gel electrophoresis
RT-qPCR	Reverse transcription-quantitative polymerase chain reaction
