# Supplementary material for: Discovery of Putative Phosphorylation Sites in Cre1 and Ace1 as Engineering Targets for Enhanced Cellulase Production in Trichoderma reesei
Source: Int J Mol Sci. 2026 Oct 15;27(20):9145. doi: 10.3390/ijms27209145 (PMC13704899; doi:10.3390/ijms27209145)
Supplement: Supplementary file 1 [file ijms-27-09145-s001.zip › ijms-4584630-supplementary.pdf]

Supporting Information for

Discovery of Putative Phosphorylation Sites in Cre1 and Ace1 as Engineering Targets for Enhanced Cellulase Production in *Trichoderma reesei*

Jiahui Liu, Sunxing Wang, Xin Gao, Yumeng Chen and Wei Wang *

State Key Lab of Bioreactor Engineering, East China University of Science and Technology, Shanghai 200237, China

* Correspondence: wadexp@ecust.edu.cn or wadexp@163.com; Tel.: +86-021-64251907

Table S1. Information of phosphorylation sites identified in this study.

Protein	A0A024SID9	G0RB08	G0RB08	G0RB08	G0RB08
Proteins	A0A024SID9; G0RCC6	G0RB08|G0RB08_HYPJQ	G0RB08|G0RB08_HYPJQ	G0RB08|G0RB08_HYPJQ	G0RB08|G0RB08_HYPJQ
Gene Name	ace1	cre1	cre1	cre1	cre1
Protein Name	A0A024SID9_HYPJR;G0RCC6_HYPJQ	Carbon catabolite repressor protein	Carbon catabolite repressor protein	Carbon catabolite repressor protein	Carbon catabolite repressor protein
Description	C2H2-type domain-containing protein (Fragment)	Carbon catabolite repressor protein OS=Hypocrea jecorina (strain QM6a) OX=431241 GN=cre1 PE=4 SV=1	Carbon catabolite repressor protein OS=Hypocrea jecorina (strain QM6a) OX=431241 GN=cre1 PE=4 SV=1	Carbon catabolite repressor protein OS=Hypocrea jecorina (strain QM6a) OX=431241 GN=cre1 PE=4 SV=1	Carbon catabolite repressor protein OS=Hypocrea jecorina (strain QM6a) OX=431241 GN=cre1 PE=4 SV=1
Modified peptide	NHAS(+79.97)DSGIGSSVVSTNDK	ETLS(+79.97)GGPSNHNSR	SS(+79.97)TTGS(+79.97)LAGGDLMDR	SS(+79.97)TTGS(+79.97)LAGGDLMDR	SST(+79.97)TGSLAGGDLMDR
Sequence	RTRNHASDSGIGS	IERETLSGGPSNH	PNSGRSSTTGSLA	RSSTTGSLAGGDL	NSGRSSTTGSLAG
Amino acid	S	S	S	S	T
PTMLocation	S145	S204	S388	S392	T389
Motif Sequence	RTRNHASDSGIGS	IERETLSGGPSNH	PNSGRSSTTGSLA	RSSTTGSLAGGDL	NSGRSSTTGSLAG
QM6a-1	1369.7771	1537.236237	1871.768912	1526.223415	1725.002305

QM6a-2	1887.405518	1305.365144	1776.324116	1245.986575	1215.442145
QM6a-3	699.2751465	1507.985612	1152.695663	1427.775696	1559.868324
Snf1-1					
Snf1-2					
Snf1-3					
Identified or Quantified peptide	Quantified	Quantified	Quantified	Quantified	Quantified

Table S2. Plasmids and strains constructed in this study.

Plasmid	Mutants
pcre1S204A	TrCre1S204A
pcre1S204D	TrCre1S204D
pace1S145A	TrAce1S145A
pace1S145D	TrAce1S145D

Table S3. Primers used for plasmid construction and strain verification.

Primer	Sequence
Cre1-1	ATTACGAATTCTTAATTAAATGCAACGAGCACAGTCTGCC
Cre1S204A-1R	TGGACGGCCCGCCGGCAAGCGTCTCGCG
Cre1S204D-1R	TGGACGGCCCGCCGTCAAGCGTCTCGCG
Cre1S204A-2F	CGCGAGACGCTTGCCGGCGGGCCGTCCA
Cre1S204D-2F	CGCGAGACGCTTGACGGCGGGCCGTCCA
Cre1-2	CATTATACGAAGTTATTCTAGACTACATCCGATCCATGAGGTCGCC
Cre1-3	ACTAGTGAGCTCATTTAATGTCCGGTACTCATGGCGCGCATCG
Cre1-4	AGTGCCAAGCTTATTTACGGGTCCCACGGATGCACCGCCTGC
Cre1-CF	CACTCGCTTCTCTGGGCTCTC
Cre1-CR	CCGCGTCTTACGATATGTGAGC
D70-4	TCGGACTTGCGGAGGATGTTGTAT
Hg3.6	TGCCTAGTGAATGCTCCGTAACA
Ace1-1	ATTACGAATTCTTAATTAACCAGTCCGTGTAACGCAAAGTA
Ace1-2	CATTATACGAAGTTATTCTAGAACGGAGTACGGAGTAGTACCTG
Ace1-3	ACTAGTGAGCTCATTTTTTCCTGTGCTGCACACGCC
Ace1-4	AGTGCCAAGCTTATTTGTCAGAAGTCGACTTACCGGTGCC
Ace1S145A-3R	TGCCGCTGTCGGCGGCGTGGTTT
Ace1S145A-4F	AAACCACGCCGCCGACAGCGGCA
Ace1S145D-3R	TGCCGCTGTCGTCGGCGTGGTTT
Ace1S145D-4F	AAACCACGCCGACGACAGCGGCA
Ace1-CF	CTCAGTTGGTACCTGGTAATAGGT
Ace1-CR	GGTGAGATATTCGACTGTCGCTTG

Table S4. Primers used for quantitative real-time PCR (qRT-PCR).

Primer	Sequence
Qsar1-1	TGGATCGTCAACTGGTTCTACGA
Qsar1-2	GCATGTGTAGCAACGTGGTCTTT
Q*cbh1*-1	CTCCATCTCCGAGGCTCTTACC
Q*cbh1*-2	GCAAGTGCCGCCATATCTGTTAT
Q*cre1*-1	ACTCCTACTCGTCCTTTGTCA
Q*cre1*-2	GGAACACCCTGGCCAAAGTAA
Q*ace1*-1	TGGCCTGTCTCTTAAGACTACCAT
Q*ace1*-2	TGTTCGCGGATGCGGTTGACAG
Q*ace3*-1	GCCAAGTGCGAGTACCTCAG
Q*ace3*-2	GCTGGTCGCTCTTCTTCCTC
Q*egl1*-1	GCAGCCTCACCATGAACCAGTA
Q*egl1*-2	CACCGTCAGAGTCCAGGAGATAC
Q*xyr1*-1	CTTCCTCCTCCTGCTCATCG
Q*xyr1*-2	TCGTGTGCCCTAACAATGGTC

Figure S1

(**a**)

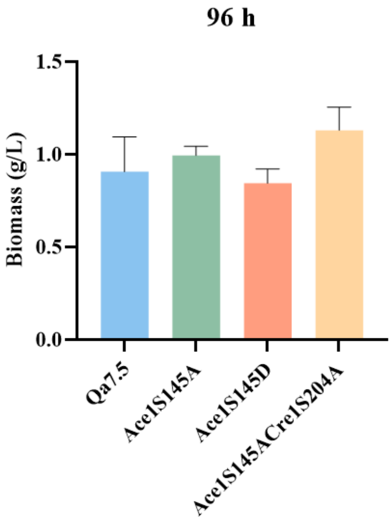

(**b**)

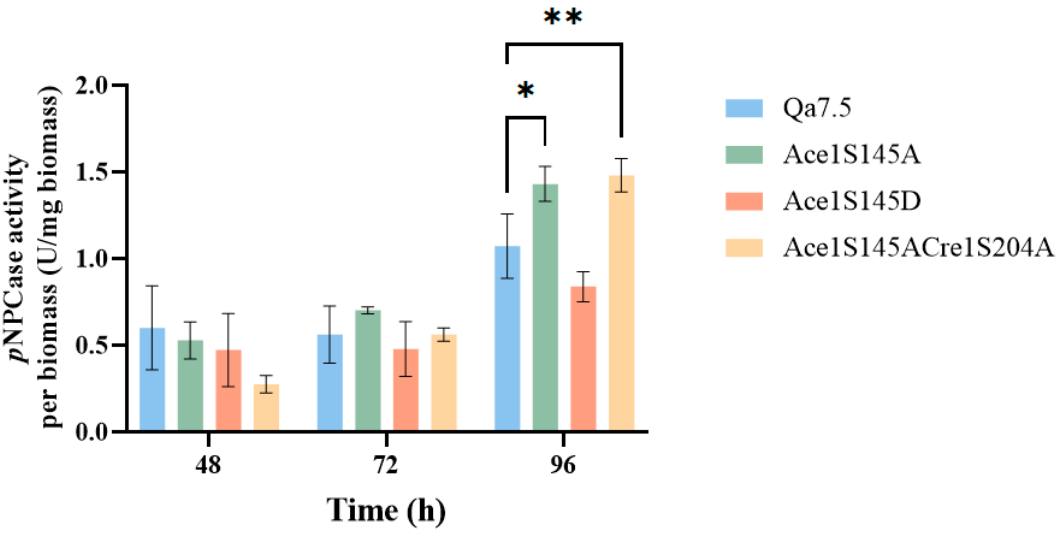

Figure S1. Biomass and biomass-normalized *p*NPCase activity of T. reesei Qa7.5 and Ace1/Cre1 phosphorylation-site mutant strains. (**a**) Estimated dry biomass of Qa7.5, Ace1S145A, Ace1S145D, and Ace1S145A/Cre1S204A after 96 h of cultivation. Biomass was estimated from intracellular protein content, assuming an average intracellular protein content of 0.32 g per gram of dry cell mass. (**b**) *p*NPCase activity normalized to the corresponding biomass at 48, 72, and 96 h. Data are presented as the mean ± SD of three independent biological replicates. Statistical significance was assessed by one-way ANOVA followed by Tukey's multiple-comparisons test ($^{**}p < 0.01$, $^{*}p < 0.05$).

Figure S2.

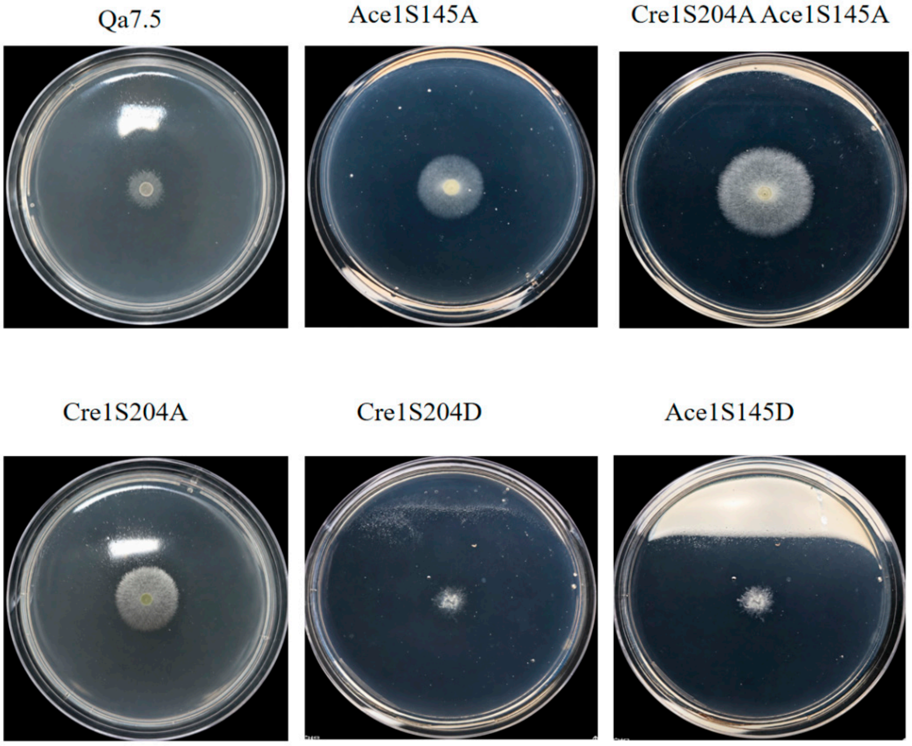

Figure S2. Colony phenotypes of the parental and mutant strains under 2-DG-containing conditions.

Qa7.5, the Cre1 and Ace1 single-mutant strains, and the Cre1/Ace1 double-mutant strains were grown on minimal medium (MM) supplemented with 1% (w/v) CMC-Na and 1.5 mM 2-deoxy-D-glucose (2-DG) at 28 °C for 72 h.

Figure S3.

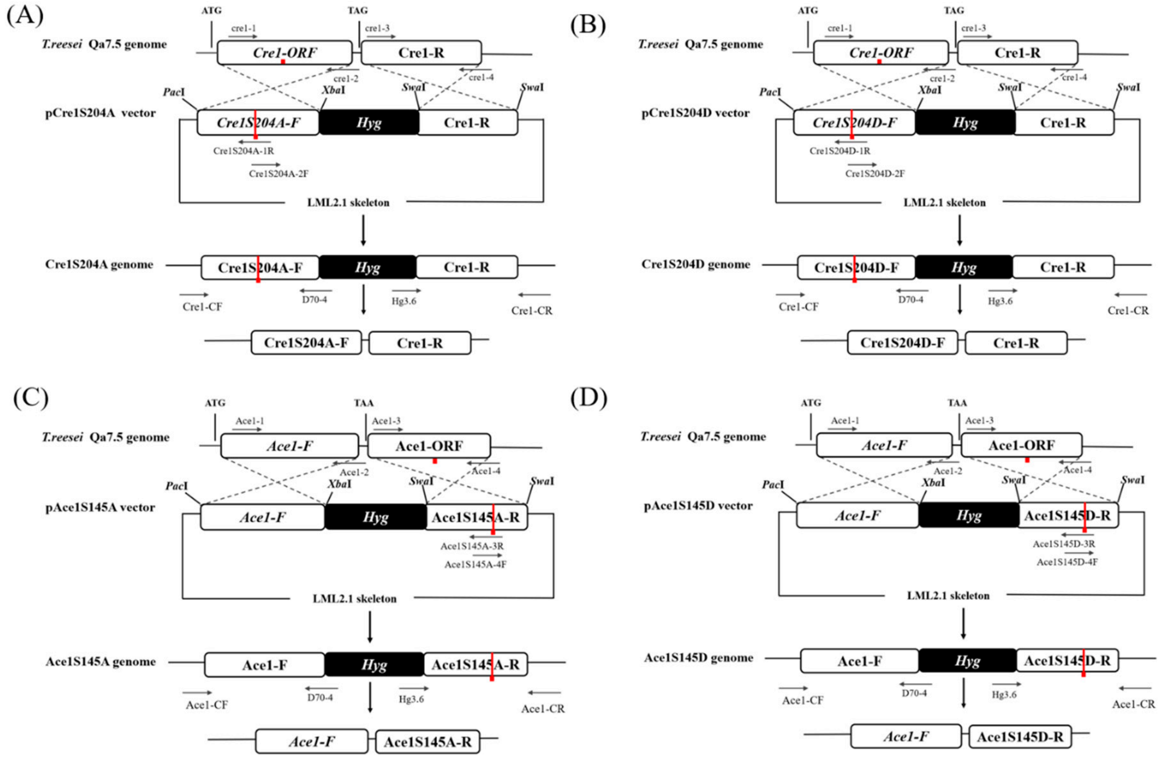

Figure S3. Construction of Cre1S204A, Cre1S204D, Ace1S145A, and Ace1S145D mutant strains. (A) The skeleton schematic diagram of Cre1S204A mutation plasmids and strain construction. (B) The skeleton schematic diagram of Cre1S204D mutation plasmids and strain construction. (C) The skeleton schematic diagram of Ace1S145A mutation plasmids and strain construction. (D) The skeleton schematic diagram of Ace1S145D mutation plasmids and strain construction.
